# Microbial Musings – October 2020

**DOI:** 10.1099/mic.0.000989

**Published:** 2020-10-29

**Authors:** Gavin H. Thomas

**Affiliations:** ^1^​ Department of Biology, University of York, York, UK

We start October thinking about change – you will have seen that we have been advertising for new editors and senior editors, and we will have some exciting new announcements coming in November’s Microbial Musings about new community-led topic areas we are launching as we start to build activities around the journal's 75th Anniversary in 2022. The *Microbiology* publishing team has also had a shuffle with Hilary Logan (@tallscientist), our journal development editor, moving to lead an exciting new project with our sister journal *Access Microbiology* – best of luck Hilary and thanks for all your hard work on the journal. We welcome Hebba Beech (@hebba_beech), who adds *Microbiology* to her journal portfolio within the Society and will be the primary contact for all things *Microbiology* going forward. Change is also happening across the pond, with the retirement of the legendary Tom Silhavy from his Editor-in-Chiefship of the American Society of Microbiology’s *Journal of Bacteriology* (JB), a position he has held since 2011. His ‘retirement’ article, entitled ‘Time to go’, is well worth a read and reflects on the massive changes in the scientific publication process and competition with other ‘for-profit’ providers in the age of open access [1]. The move away from paper is a change still within recent memory – when I was a new lecturer in the early noughties I would know immediately what was inside when the characteristic white and green glossy envelopes appeared in my mailbox with a printed copy of a paper to review attached. Memorable also as it would be printed on ‘letter’-sized paper, which was always a funny thing for people outside of the Americas.

Our first paper this month, from the group of *Microbiology* editor Nick le Brun (@Nick_Le_Brun), at the University of East Anglia, UK, concerns the maturation of nitrous oxide reductase (N_2_OR), a key enzyme in the bacterial denitrification pathway [2]. The enzyme catalyses the last reaction in the process by converting N_2_O to dinitrogen gas and, like many enzymes in this pathway, is dependent on metal cofactors for activity, in this case copper. Complex metal-containing enzymes often require additional gene products for the correct insertion of the metal and activation of the metal-bound form. In this work the authors examine a gene, *nosX*, that sits within the *nos* gene cluster that encodes the N_2_OR in *Paracoccus denitrificans *. When the gene is inactivated on the chromosome, the resulting bacteria are totally unable to reduce N_2_O, so the authors conclude that the gene is essential for some process in the maturation of the enzyme. However, when they check the copper centres they are intact and in fact when the enzyme is purified it is functional *in vitro*, suggesting that some specific *in vivo* function has been lost. This, they hypothesize, based on the work from an orthologue in *Pseudomonas stutzeri* called AbpE [3], is through the maturation of another protein, NosR, which is required for electron transfer to the N_2_OR reductase, which would explain why the enzyme is fine *in vitro* using artificial electron donors, but cannot work at the membrane *in vivo*. AbpE is thought to work by inserting the flavin adenine dinucleotide (FAD) cofactor into NosR, which, interestingly, occurs in the periplasm, which is where NosX Is located, leaving the question of how this the FAD is exported from the cytoplasm for future studies.

Our next paper sticks with the pseudomonads and is an important overturning of a long-held dogma. The work, from Laura Nolan (@LauraNolanLab), Lyne Turnbell (@lynnet3) and other members of Cynthia Whitchurch’s lab (@Cwhitch) at the iThree Institute (@ithreeinst), University of Technology Sydney, Australia, and now the Quadram Institute (@TheQuadram) in Norwich, UK, looks to understand more about the biology of *Pseudomonas aeruginosa* when growing in biofilms and reveals a fundamentally important insight into its genetic mechanisms [4]. Noting that *P. aeruginosa* genomes contain genes that would appear to encode the machinery required for natural transformation, and undaunted by the accepted idea that *P. aeruginosa* is not naturally competent [5], the first thing the authors do is demonstrate that under biofilm-like conditions they can clearly detect genetic transformation. This is also seen on plates in colony biofilms, and while the rates of transformation vary within strains, the lab strain PAO1 has this ability, which is also detectable, but at a much lower frequency, in another common lab strain, PA14. The authors then go on to show that this phenotype can also be seen in static broth cultures of *P. aeruginosa *. Finally, they look a little at the mechanism and demonstrate that the type IV pili appear to be important, but not essential, for transformation to occur. The paper is important as it promotes a rethink on how this pathogen is able to adapt through the acquisition and recombination of exogenous DNA into its chromosome, which could clearly be important for its evolution of virulence and antibiotic resistance traits. Further, this is Professor Whitchurch’s second paper this year in *Microbiology* – perhaps some kind of a prize is in order?

Sticking with biofilms, we turn to a paper from the group of Bodo Philipp from Westfälische Wilhelms-Universität Münster, Germany, who study a genus of bacteria, *Sphingomonas *, that are associated with bio-fouling through biofilm formation on pure-water systems [6]. Working with a strain isolated from a water-fitration membrane, *Sphingomonas* sp. strain S2M10, they use a transposon-based mutagenesis approach to isolate mutants that cannot form biofilms and find mutations that either reduce the synthesis of one of the surface glycans produced by this genus, the acidic polysaccharide sphingan, or have reduced flagellar motility [7]. By manipulating the regulation of sphingan biosynthesis using a particular histidine kinase mutant, they observe much higher levels of cell aggregation. When they grow the wild-type bacteria under different nutritional conditions, which they know regulate sphingan biosynthesis, they see corresponding changes in the level of biofilm formation, suggesting that the physiological triggers for this process on water filters may be nutritional and act by turning on expression of this complex extracellular polymer.

Following on from this paper finding roles for flagella in attachment is the Editor’s Choice for this month, selected by Senior Editor Hana Sychrova. This is an interesting new paper from Eliza Wolfson (@eliza_coli) from the lab of Dave Gally (@strain9000) in collaboration with Ariel Blocker’s group in Bristol (@ArielBlocker) [8]. The paper will be described in the Society’s blog pages in more detail, but it sounds like the end of a long story where the authors have now demonstrated that flagella are important for initial binding to host cell membranes and to the underlying cytoskeletal proteins during the process of intimate attachment during cell invasion in pathogenic *Escherichia coli* strains and *Salmonella enterica* serovar Typhimurium, respectively. Check out first author Eliza’s other work too, as she is also a talented scientific illustrator (https://lizawolfson.co.uk/).

Finally, when a paper from one’s own group is published in the issue, then rather more insight than usual into the process can be presented [9]. This paper, although only a Short Communication, took a long time to come about and was triggered by a publication in 2010 discovering a new ppGpp-induced protein in *S. enterica* serovar Typhimurium involved in host colonization and specifically intracellular survival in macrophages [10]. The authors didn’t know what the protein did, but for me this was exciting, as it was a component of one of a small number of tripartite ATP-independent periplasmic (TRAP) transporters from enteric bacteria that we had been thinking of studying to examine their substrate specificity. These transporters in Gram-negative bacteria have a soluble substrate-binding protein (SBP) that defines the substrate specificity of the uptake system and can usually be readily expressed and purified in *E. coli *. Hence, I persuaded my grad student Abbas Maqbool (@abbasmaqbool2), who was by now an expert in characterizing SBPs [11], to work with Amna Afzal, to clone, express and purify the protein, which they together quickly achieved in 2012. From our previous work on TRAP transporters, we had a pretty good idea what we thought the substrate was, a hexuronic acid, but Abbas could not see any binding using our usual methods. Abbas left for a postdoc at the Sainsbury lab in Norwich, UK, and years passed. A few years later another grad student, Konstantinos Drousiotis (@Ecolinnit), briefly re-prepped the protein, but again could see no evidence of binding. More years passed. A talented integrated Master’s student joined the lab and we sent him out to finally crack this nut. We had not used one of our old tricks in the past, which was to denature and refold the protein on the column before doing binding experiments, to remove any endogenous bound substrate. When the student, Cavan Bennett-Ness (@I_am_Cavan), did this with my research assistant Reyme Herman (@ReymeHerman), and we got onto the fluorimeter, lo and behold we observed binding for d-glucuronate that was saturable and specific. Cavan finished his project, getting onto an excellent grad school programme in Edinburgh for his PhD, and Reyme then repeated it all and used other biophysical methods available in Andrew Leech’s molecular interactions lab at York to support the initial findings. Reyme also used one of our favourite tools, MicrobesOnline [12], from the Arkin lab at Berkeley, USA, where we discovered orthologues for the *Salmonella* protein encoded in full hexuronic acid catabolic gene clusters, further supporting the experimental data, and he then confirmed that *Salmonella* can use d-glucuronate as a sole carbon source. Anyway, the question was answered, this protein that is important for macrophage survival forms part of a TRAP transporter for d-glucuronic acid, strongly suggesting that the uptake and catabolism of this acid sugar is important for intracellular growth, but grad students be warned when your supervisor suggests a ‘quick side project’!
